# Complete Genome Sequence of the *Escherichia coli* PMV-1 Strain, a Model Extraintestinal Pathogenic *E. coli* Strain Used for Host-Pathogen Interaction Studies

**DOI:** 10.1128/genomeA.00913-13

**Published:** 2013-10-24

**Authors:** Francesc Peris-Bondia, Eric Muraille, Laurence Van Melderen

**Affiliations:** Laboratoire de Génétique et Physiologie Bactérienne, IBMM, Faculté des Sciences, Université Libre de Bruxelles (ULB), Brussels, Belgiuma; Laboratoire de Parasitologie, Faculté de Médecine, Université Libre de Bruxelles (ULB), Brussels, Belgiumb

## Abstract

*Escherichia coli* is a highly versatile species, causing diverse intestinal and extraintestinal infections. Here, we present the complete genome sequence of PMV-1, an O18:K1 extraintestinal pathogenic *E. coli* (ExPEC) strain that is used as a model for peritonitis in mice and was useful for deciphering the innate immune response triggered by ExPEC infections.

## GENOME ANNOUNCEMENT

*Escherichia coli* causes a broad spectrum of disease that relies on the acquisition of specific virulence factors, which confer an increased ability to adapt to new niches. In that context, the genome of the *E. coli* PMV-1 strain and its plasmids were sequenced. This strain is an appropriate model for host-pathogen interactions since it has been proven to be highly virulent after intraperitoneal administration in mouse models (1, 2). The whole genome was sequenced to a depth of 40× coverage using pyrosequencing by Macrogen Inc. *E. coli* strain UTI89 was used as a backbone to perform the assembly by MIRA (3) and the Staden package (4). Phylogenetic analysis was inferred using ClonalFrame (5) with three iterations between samples and representative genomes of *E. coli*. Protein-coding sequences (CDSs) and noncoding RNAs (ncRNAs) were identified using Prokka (6) and were compared with Glimmer (7) results. The functional annotation of the CDSs was performed using BLASTp (8) against the nr protein database (9) and VFDB (10). Genes for tRNAs, transfer-messenger RNA (tmRNA), rRNAs, and other small RNAs were identified using the Rfam database (11) and Infernal (12). In order to identify the genomic islands (GIs), a reciprocal best-hit blast with ≥70% identity was performed versus *E. coli* K-12 MG1655. GIs were defined by ≥10 consecutive CDSs that were not homologous to K-12 CDSs. Prophages were detected using PHAST (13).

The genome of PMV-1 comprises a circular chromosome of 4,984,940 bp and two plasmids of 98,864 bp and >127,123 bp (in two contigs of 83,288 and 43,835 bp). Phylogenetic analysis identified *E. coli* IHE3034, an extraintestinal pathogenic *E. coli* (ExPEC) O18:K1 strain (14), as the closest whole-genome-sequenced strain. The 98-kb plasmid is similar to the IncI pHUSEC41-1 plasmid (15) and comprises 106 open reading frames (ORFs). It lacks a 7,237-bp region flanked by transposase- and resolvase-encoding genes containing predicted β-lactamase, streptomycin phosphotransferase, and aminoglycoside resistance genes. This region is replaced by an IncQ incompatibility system, arsenic resistance, phosphoglucosamine mutase, and sulfonamide resistance genes. The other two scaffolds are part of an incomplete sequenced plasmid similar to pECOS88 (16). This IncFI-IncFII plasmid is associated with neonatal meningitis cases and harbors iron uptake systems (aerobactin, salmochelin, and *sitA*). Thirty-seven GIs of >5 kb carrying virulence factors, such as genes for S-fimbriae and iron acquisition systems and capsule-encoding genes, as well as a putative type VI secretion system (T6SS) and several predicted *hcp* genes (17), were identified. In addition, 5 GIs containing antibiotic resistance genes (encoding β-lactamase, efflux pumps, and multidrug transporters) were detected. Among the 9 prophages that were identified, 3 carry virulence factors, an iron-manganese transport system (*sit*), a putative *O*-acetyltranferase (*neuO*) (18), and type 1 fimbriae (*fim*). Despite the cytotoxic necrotizing factor 1 (CNF1) known to be involved in blood-brain barrier crossing (19), this strain carries all the virulence factors responsible for meningitis and sepsis (20).

### Nucleotide sequence accession numbers.

The annotated genome sequences of *E. coli* PMV-1 have been deposited in public databases under the accession no. HG428755 for the complete genome and under accession no. HG428756 and CBTO010000001 to CBTO010000002 for *E. coli* PMV-1 plasmids pHUSEC41-1-like and pECOS88-like, respectively.
